# Liver Embolization for Subcapsular Hematoma in HELLP (Hemolysis, Elevated Liver Enzymes, and Low Platelets) Syndrome: A Case Report

**DOI:** 10.7759/cureus.102839

**Published:** 2026-02-02

**Authors:** El Hajjami Ayoub, Bouktib Youssef, Badr Boutakioute, Meriem Ouali Idrissi, Najat Cherif Idrissi El Ganouni

**Affiliations:** 1 Radiology Department, Arrazi Hospital, Mohammed VI University Hospital, Cadi Ayyad University, Marrakesh, MAR

**Keywords:** eclampsia, hellp syndrome, hepatic infarction, interventional radiology, liver rupture, maternal morbidity, minimally invasive management, postpartum complication, subcapsular hepatic hematoma, transarterial embolization

## Abstract

HELLP syndrome is a severe obstetric complication characterized by hemolysis, elevated liver enzymes, and low platelet count, typically occurring in the third trimester or postpartum period. One of its rare but life-threatening complications is subcapsular hepatic hematoma, which may rupture and cause catastrophic hemorrhage. We report the case of a 38-year-old woman with type 2 diabetes mellitus who developed severe right upper quadrant pain three days after a cesarean delivery complicated by eclampsia. Laboratory findings were consistent with HELLP syndrome. Abdominal ultrasound and contrast-enhanced computed tomography (CT) revealed a right subcapsular hepatic hematoma measuring 34 mm in thickness, associated with hepatic infarctions and moderate ascites. Given the high risk of rupture, urgent transarterial embolization was performed via selective catheterization of the right hepatic artery using 400-micron microparticles, achieving complete hemostasis while preserving hepatic perfusion. The patient remained stable and recovered uneventfully, with gradual resolution of the hematoma and normalization of laboratory parameters, thereby avoiding surgical intervention. This case highlights the importance of early recognition and prompt interventional management of hepatic complications in HELLP syndrome. Transarterial embolization offers a minimally invasive, effective, and organ-preserving alternative to surgery in selected hemodynamically stable patients, underscoring the value of multidisciplinary collaboration among obstetric, critical care, and interventional radiology teams to optimize maternal outcomes.

## Introduction

HELLP syndrome is a severe obstetric complication characterized by hemolysis, elevated liver enzymes, and low platelet count, often developing in the third trimester of pregnancy or in the postpartum period [1]. It can be seen in approximately 0.5%-0.9% of all pregnancies and in 10%-20% of patients with severe preeclampsia [1]. Diagnosis can be challenging due to its variable and often non-specific clinical presentation, frequently overlapping with other hypertensive disorders of pregnancy [2]. A particularly life-threatening complication of HELLP syndrome is subcapsular liver hematoma, which can rupture and lead to catastrophic hemorrhage and maternal mortality, historically reported at around 17%-59%, particularly when rupture occurs [2]. While most reported cases of ruptured hepatic subcapsular hematoma are managed surgically, transarterial embolization has emerged as a less invasive yet effective therapeutic option in select cases [2]. We report a case of HELLP syndrome complicated by a ruptured subcapsular liver hematoma successfully managed with transarterial embolization, emphasizing a multidisciplinary approach for optimal patient outcomes [1].

## Case presentation

A 38-year-old woman with a medical history of type 2 diabetes mellitus was referred to the obstetric intensive care unit three days after a cesarean delivery complicated by eclampsia. She presented with severe right upper quadrant pain and malaise but no signs of hemodynamic shock. Laboratory investigations revealed severe thrombocytopenia, elevated liver enzymes, and biochemical hemolysis, consistent with HELLP syndrome in the postpartum period.

**Figure 1:**
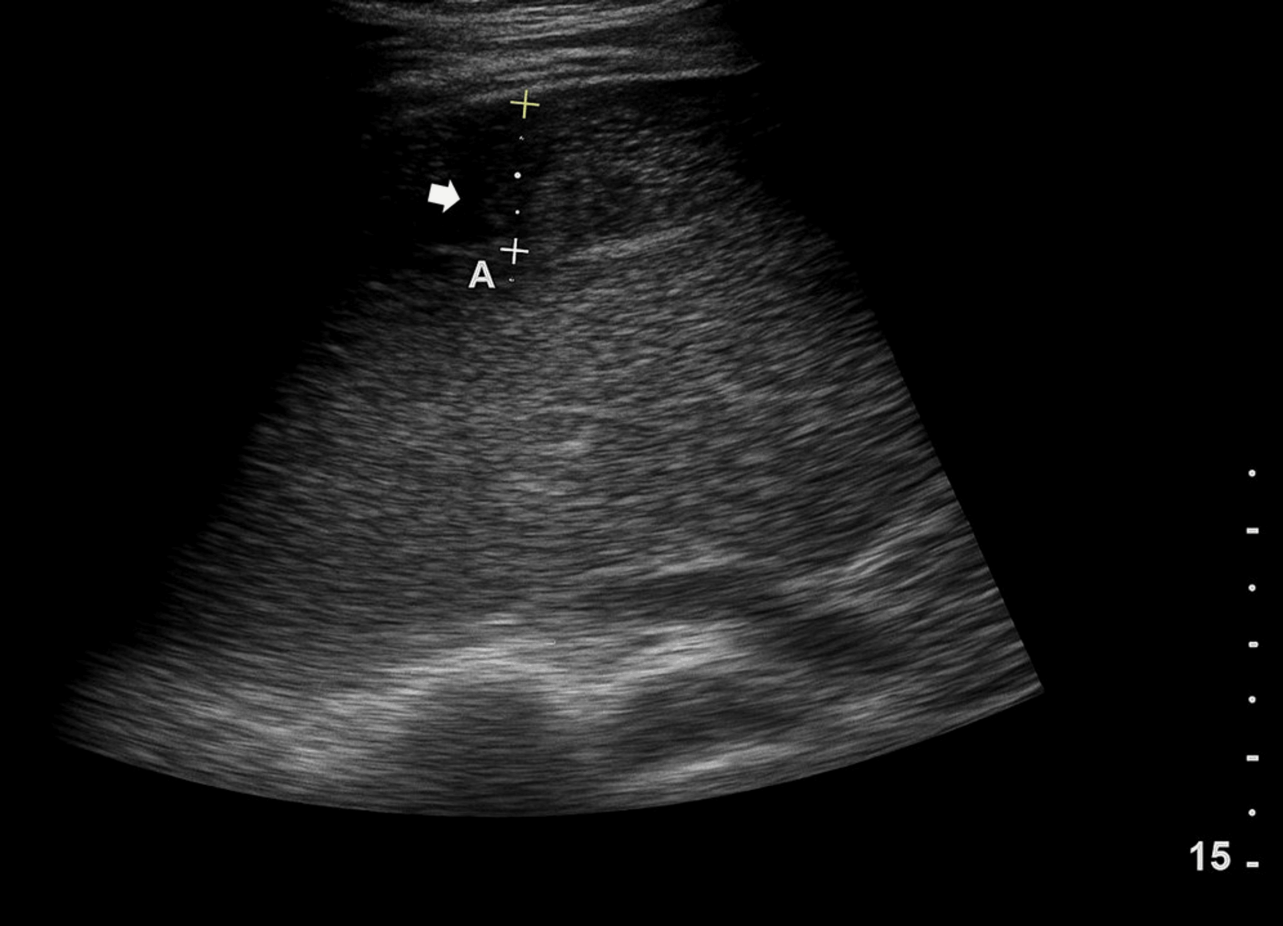
Abdominal ultrasonography showing a well-defined heterogeneous subcapsular collection (white arrow) over the anterior, posterior, and right lateral aspects of the right hepatic lobe, measuring approximately 34 mm in maximal thickness, consistent with a subcapsular hematoma.

Abdominal ultrasonography revealed a well-defined heterogeneous subcapsular collection overlying the anterior, posterior, and right lateral aspects of the right hepatic lobe, measuring approximately 34 mm in maximal thickness, consistent with a subcapsular hematoma (Figure 1).

**Figure 2:**
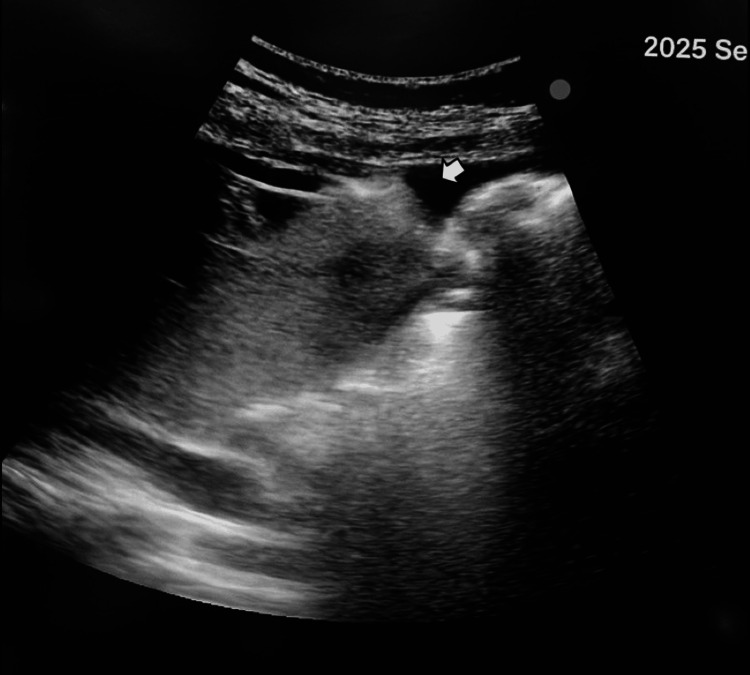
Ultrasound image demonstrating moderate anechoic peritoneal effusion (white arrow) in the hepatorenal recess, consistent with reactive or hemorrhagic ascites.

The adjacent liver parenchyma appeared compressed with mild capsular bulging, and several poorly demarcated hypoechoic areas were identified within the right hepatic lobe, suggestive of hepatic infarctions. A moderate amount of free anechoic peritoneal fluid was also visualized in the hepatorenal recess, perisplenic space, and pelvis, consistent with reactive or hemorrhagic ascites (Figure 2).

The gallbladder and biliary tree appeared unremarkable, with no intrahepatic biliary dilatation or focal hepatic mass detected. Color Doppler evaluation demonstrated preserved main hepatic arterial and venous flows without evidence of pseudoaneurysm, thrombosis, or arteriovenous shunting.

**Figure 3:**
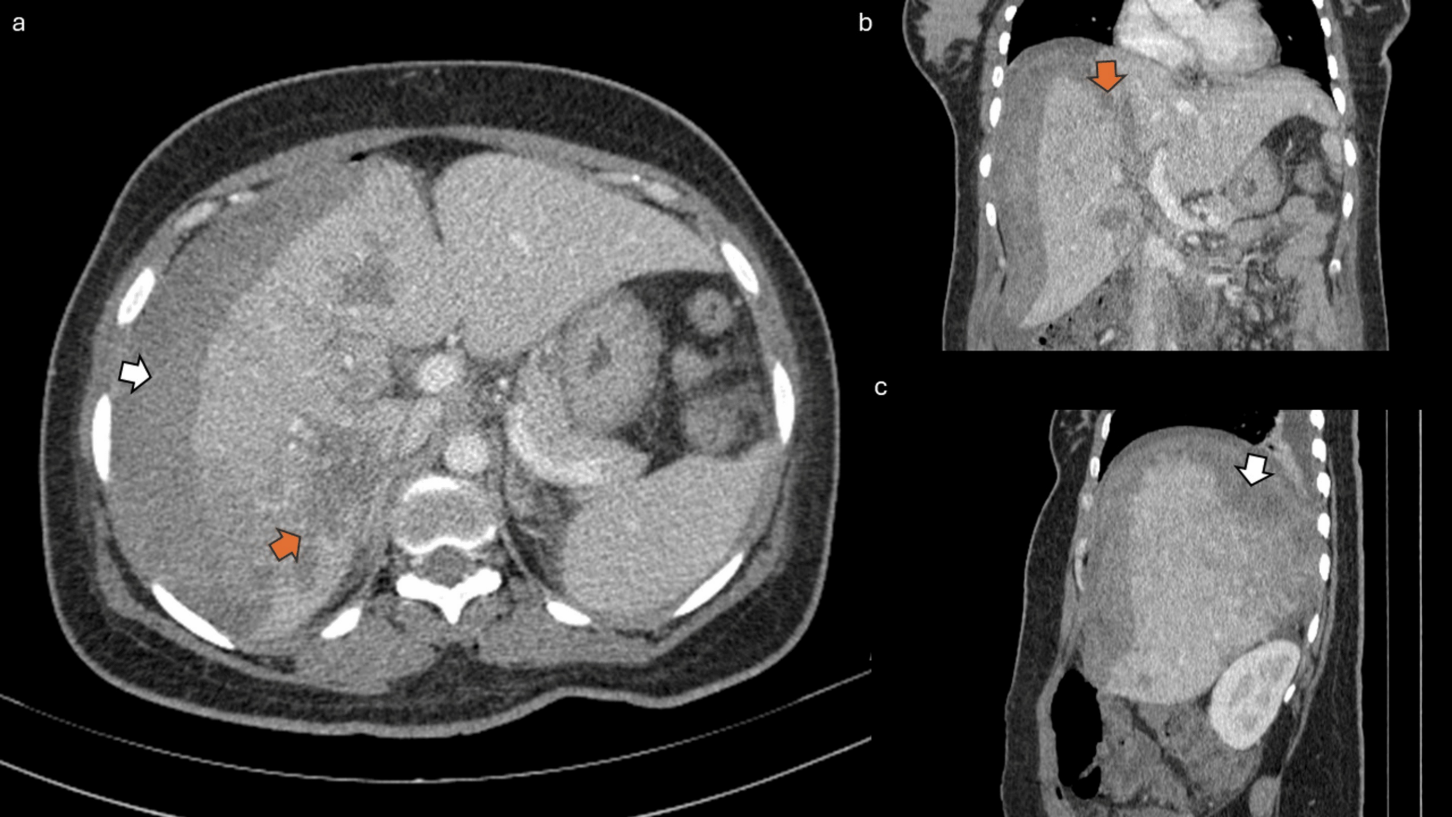
Contrast-enhanced CT scan of the abdomen in axial (a), coronal (b), and sagittal (c) views revealing a heterogeneous subcapsular hepatic hematoma (white arrows) over the right hepatic lobe and multiple poorly defined hypodense areas of hepatic infarction (orange arrows). CT: computed tomography

Contrast-enhanced computed tomography of the abdomen and pelvis confirmed a heterogeneous hyperdense subcapsular hepatic hematoma on the right, measuring 34 mm in maximum thickness, without enhancement after contrast administration (Figure 3, white arrows).

**Figure 4:**
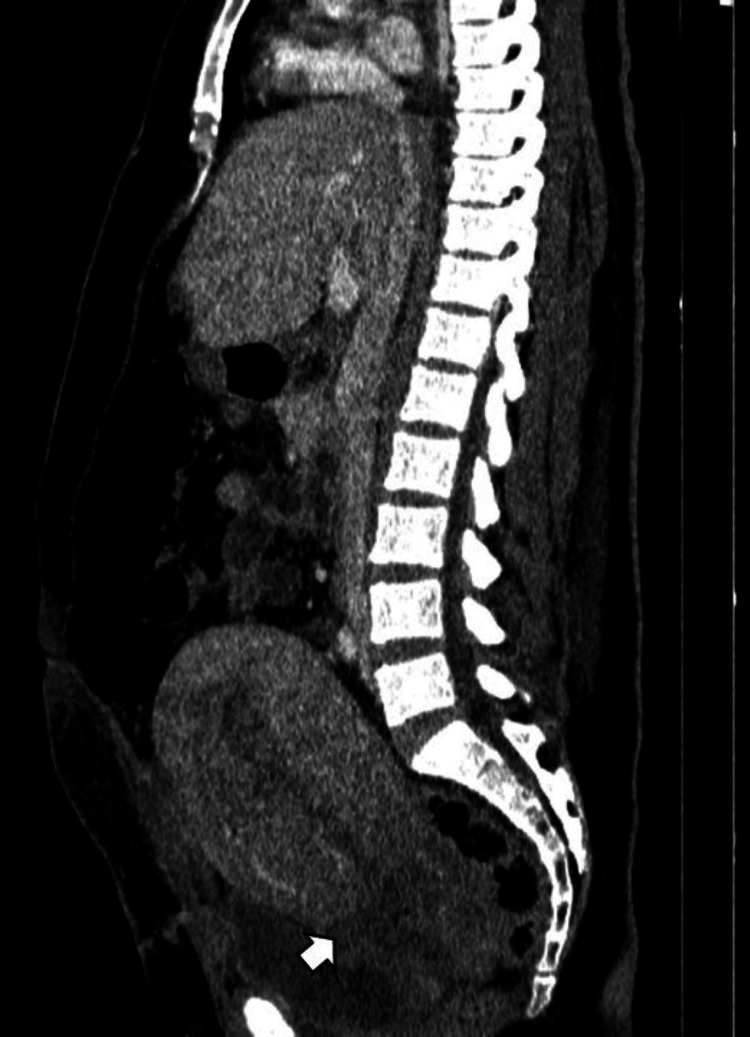
Sagittal contrast-enhanced CT image showing anterior abdominal wall edema and small postoperative air bubbles adjacent to the cesarean scar (white arrow). CT: computed tomography

Multiple poorly defined hypodense areas of hepatic infarction were also observed (Figure 3, orange arrows) in the right lobe. There was associated periportal, perivesicular, and mesenteric fat infiltration, as well as mild edema of the anterior abdominal wall with small air bubbles near the operative scar (Figure 4).

**Figure 5:**
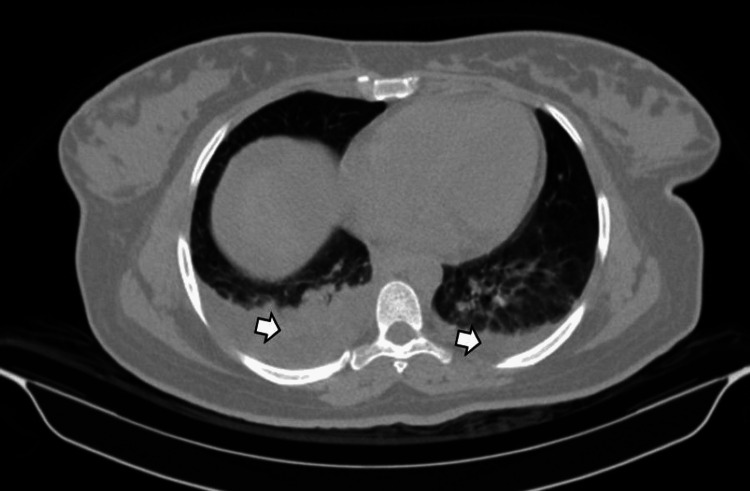
Chest CT image demonstrating bilateral pleural effusions (white arrows) with adjacent passive atelectasis. CT: computed tomography

Moderate effusion was present in the perihepatic, perisplenic, paracolic, and pelvic spaces. Bilateral pleural effusions with adjacent passive atelectasis were also noted (Figure 5).

**Figure 6:**
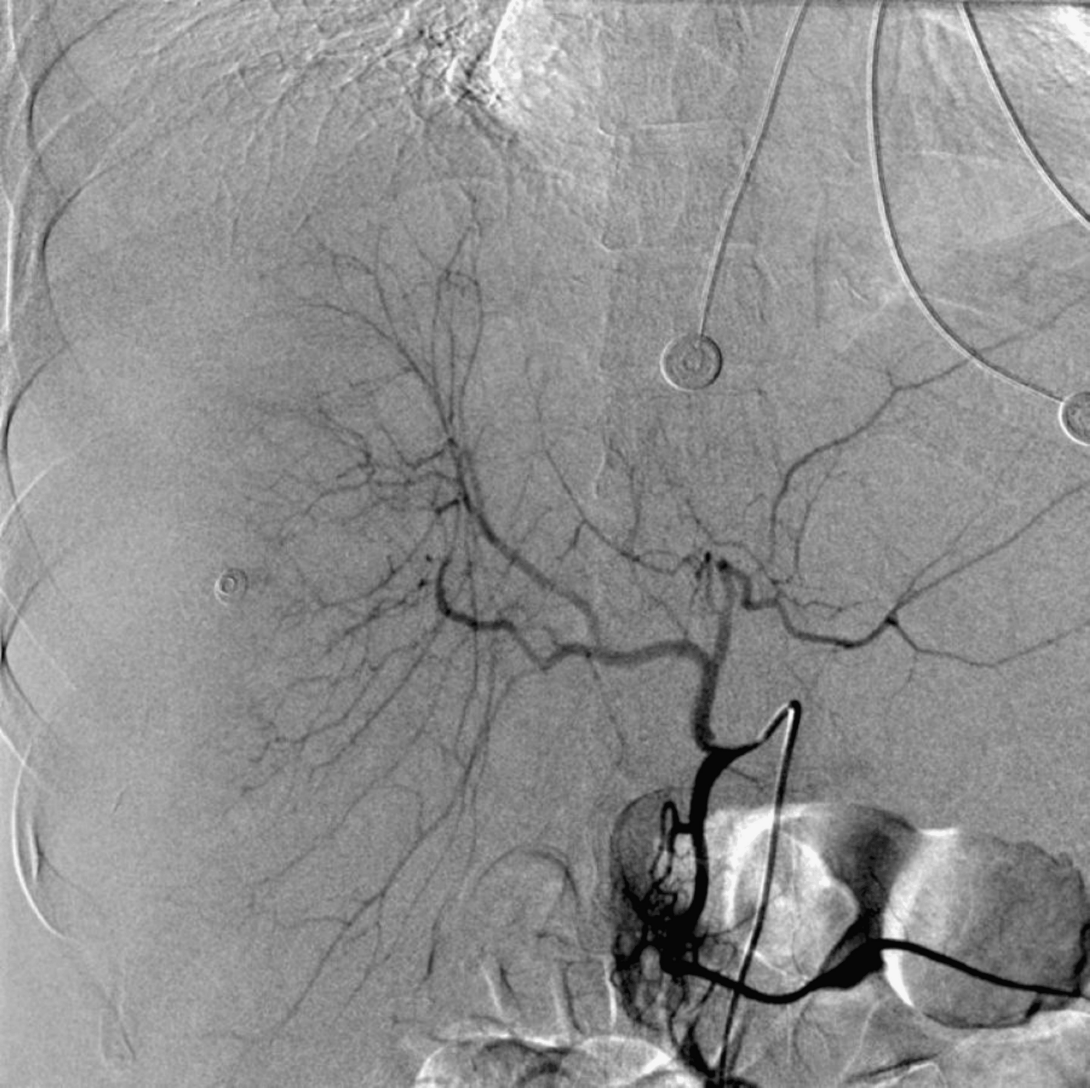
Fluoroscopic image showing selective catheterization of the proper hepatic artery using a Cobra C2 catheter with subtle vascular blush areas.

Given the high risk of hepatic rupture and hemodynamic deterioration, an urgent transcatheter hepatic artery embolization was undertaken. Vascular access was obtained via the right common femoral artery using the Seldinger technique under local anesthesia. A 5-French introducer sheath was placed, and a Cobra C2 catheter was advanced to perform selective catheterization of the celiac trunk and the proper hepatic artery under fluoroscopic guidance (Figure 6).

**Figure 7:**
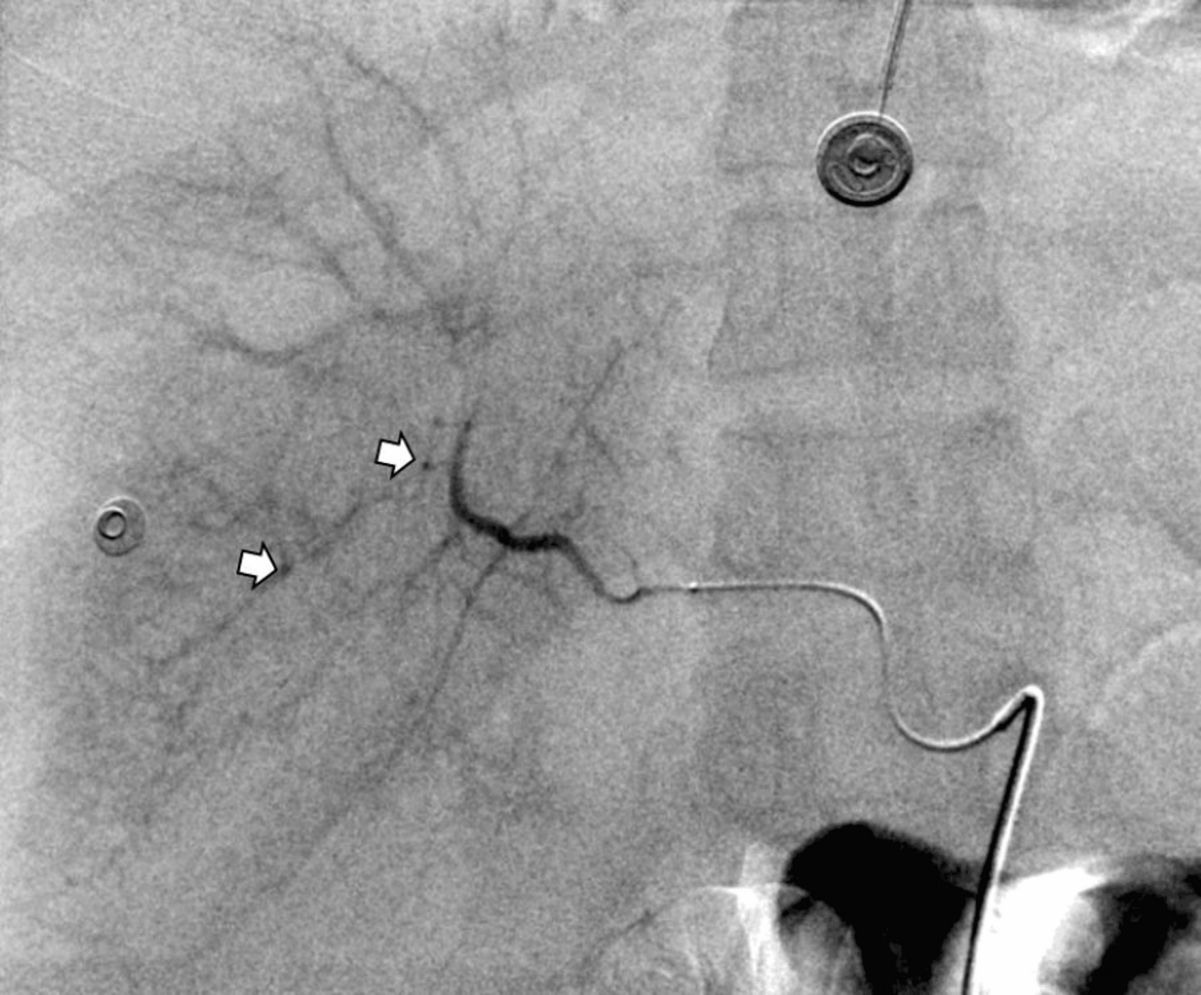
Fluoroscopic image showing selective catheterization of the proper hepatic artery using a Cobra C2 catheter with subtle vascular blush areas.

Subsequently, a 2.7-French microcatheter (Progreat®, Terumo, Tokyo, Japan) was coaxially introduced for superselective access to the right hepatic arterial branches. Angiography revealed a subtle focal vascular blush, confirming the site of arterial bleeding (Figure 7).

Embolization was carried out using 8 mL of 400-micron HydroPearl® microparticles (Terumo) until complete stasis of the targeted vessels was achieved. A final angiographic control confirmed cessation of extravasation and preservation of distal perfusion to unaffected hepatic territories. The procedure was completed without periprocedural complications in two hours, and hemodynamic stability was maintained throughout.

Postoperatively, the patient was monitored in the intensive care unit with hemodynamic stabilization, transfusion support, and correction of coagulopathy. Serial imaging and laboratory follow-up demonstrated progressive improvement with partial resorption of the hematoma and normalization of liver enzymes and platelet count. The patient recovered without the need for surgical intervention and was discharged in stable condition.

## Discussion

Subcapsular hepatic hematoma in the setting of HELLP syndrome is a rare but serious complication occurring in approximately one in 100,000 pregnancies, often necessitating prompt and aggressive management due to its high maternal and fetal mortality rates [2]. Early detection and intervention are paramount for improving outcomes, as delayed management can lead to further complications such as intra-abdominal abscesses or pleural effusions [2,3]. Furthermore, the presence of hepatic infarction, as observed in this case, can exacerbate liver dysfunction and increase the risk of subsequent liver failure [4].

The underlying mechanism involves hepatic necrosis and rupture of the sinusoidal capillaries caused by pregnancy-induced hypertension, leading to hemorrhage within the liver parenchyma and beneath Glisson's capsule [3]. This intracapsular bleeding can expand, causing ischemic necrosis of the liver and potentially leading to capsular rupture and life-threatening hemoperitoneum [1].

Diagnosis relies on imaging modalities, with ultrasound providing initial detection and CT defining the extent, presence of rupture, and active bleeding [5,6]. Definitive identification of the bleeding site and active extravasation, crucial for guiding interventional procedures, is typically achieved through angiography [7]. In this context, transcatheter arterial embolization emerges as a minimally invasive yet highly effective treatment modality, particularly in hemodynamically stable patients, by selectively occluding the bleeding vessels [1].

Management depends on the severity of the condition, ranging from conservative observation to arterial embolization or surgery. When conservative measures fail or in cases of hemodynamic instability, surgical intervention, such as hepatic packing or liver resection, may be warranted to control bleeding and prevent further deterioration [6].

In our case, the presence of active bleeding warranted urgent embolization, which was performed successfully without complications. This approach effectively stabilized the patient, preventing the need for more invasive procedures like hepatic transplantation, which has been reported in cases where embolization contributed to worsening hepatic ischemia [1].

This technique demonstrates superior outcomes over surgical intervention, particularly for postpartum subcapsular hematoma, by minimizing blood loss and preserving liver parenchyma. The application of transcatheter arterial embolization is further supported by evidence suggesting its efficacy in achieving hemostasis and preventing rebleeding, thereby reducing the need for more aggressive surgical interventions [1].

## Conclusions

In conclusion, this case highlights the critical role of timely diagnosis and interventional radiology in managing subcapsular hepatic hematoma associated with HELLP syndrome, offering a less invasive alternative to surgical approaches with favorable outcomes. It underscores the importance of a multidisciplinary approach, integrating obstetrics, critical care, and interventional radiology, to optimize patient management. Future research should focus on refining patient selection criteria for embolization versus surgical intervention to further improve outcomes and reduce morbidity associated with this rare but severe obstetric complication. Further investigation into optimal embolization techniques, including the choice of embolic agents and the extent of vessel occlusion, is also warranted to minimize the risk of post-embolization liver injury while ensuring effective hemorrhage control.
